# Ankle-Foot Orthosis and Footwear Interactions in Adult Healthy and Patient Populations: Systematic Review of Biomechanical Outcomes

**DOI:** 10.2196/93423

**Published:** 2026-10-13

**Authors:** Alexandra Millar, Christian Greve, Juha M Hijmans

**Affiliations:** 1 University of Groningen, University Medical Center Groningen, Department of Rehabilitation Medicine Groningen The Netherlands; 2 University of Groningen, University Medical Center Groningen, Department of Human Movement Sciences Groningen The Netherlands

**Keywords:** gait, adult, orthotic devices, shoes, biomechanical phenomena, ankle-foot orthoses, AFO-FC, ankle-foot orthosis and footwear combination

## Abstract

**Background:**

Both ankle-foot orthoses (AFOs) and footwear adaptations are used to improve gait across a broad range of neuromuscular disorders. AFOs and footwear can each independently influence gait through the manipulation of certain design parameters, such as AFO footplate stiffness or the use of a forefoot rocker. While the effects of design parameters from AFOs and footwear on gait have been investigated independently, how these parameters interact to influence walking biomechanics remains poorly understood.

**Objective:**

This systematic literature review aims to provide an overview of the interacting effects between AFOs and footwear on gait biomechanics from both a theoretical standpoint and through the synthesis of existing literature.

**Methods:**

A search of existing literature was conducted across 4 major databases. Studies were eligible if they included adult participants from healthy or patient populations. Studies included should have investigated biomechanical gait outcomes recorded during walking with an AFO and at least two different footwear conditions. Quality assessment was performed using the Downs and Black checklist and AFO reporting guidelines.

**Results:**

A total of 14 studies were included in this review. Across these 14 studies, a total of 204 participants (108 healthy, 84 patients after stroke, and 12 unilateral ankle weakness or pain) were included. The majority of these studies were within-subjects designs. Footwear adaptations were primarily changes to the heel-sole differential (HSD; 7/14), followed by forefoot rockers (5/14), with some studies on changes to heel shape and hardness (3/14), and a study on mediolateral wedging. These adaptations were used in combination with primarily rigid AFOs (10/14), with studies also using dorsiflexion-free and frontal plane restrictive AFOs.

**Conclusions:**

Increasing the HSD can be used to increase knee flexion across the stance phase in rigid AFO users, including in the third rocker when HSD is manipulated between the shoe and AFO. Beyond changing the HSD, there is a lack of well-designed experimental studies exploring footwear adaptations. Detailed reporting of both AFO and footwear design choices is necessary for future work in order to make informed statements on biomechanics.

## Introduction

### Background

The ankle-foot complex plays a crucial role in human walking due to its contributions to both balance and forward propulsion [1]. Plantarflexor muscle weakness, due to neuromuscular disorders such as stroke, can disrupt the biomechanics of gait, leading to slower walking speeds and a less efficient gait pattern [2,3]. To facilitate mobility and an efficient walking pattern, ankle-foot orthoses (AFOs) may be prescribed.

There exists a wide range of AFOs in a variety of shapes, materials, and stiffnesses, with varying effects on patient gait biomechanics [4,5]. These design parameters are often used to optimize biomechanical outcomes for individual patients. For example, increasing the bending stiffness of an AFO can reduce knee flexion during the second rocker in patients with crouch gait [6]. Similarly, increasing footplate stiffness can maximize energy storage and return, reducing the energetic cost of walking [7].

Adapted footwear such as rocker shoes have been investigated independent of AFO use and shown to affect gait kinematics and kinetics [8,9]. As AFOs are typically worn with footwear, it is essential to consider how footwear design interacts with AFOs to influence gait. When combined, footwear and AFO form an integrated orthotic system where the shape and bending stiffness of both devices play a combined role in influencing how forces are transmitted through the lower limb during gait. Consequently, footwear adaptations can alter the biomechanical effects of AFOs on gait. Despite these interactions, the specific impact of different footwear parameters on AFO performance remains unclear [10]. The footwear design can either complement or counteract the intended mechanical function of the AFO. For instance, when the ankle is immobilized with a rigid AFO, increasing the heel-sole differential (HSD) can increase knee flexion during midstance [11]. Conversely, a rocker shoe with a proximal apex position may shorten stance phase duration [12] and reduce the energy storage and return of a carbon fiber AFO, potentially negating its benefits.

Although the importance of the interaction between AFOs and footwear is recognized, most existing research has focused on sagittal plane alignment, particularly the shank-to-vertical angle (SVA), and often within specific patient populations [4,10,11]. While systematic reviews have examined the biomechanical effects of footwear or AFOs independently [13,14], no systematic review to date has examined the biomechanical effects resulting from the interactions between ankle-foot orthosis and footwear combinations (AFO-FCs) in adults. The aim of this review is to provide a systematic overview of the current evidence on AFO-FCs. Specifically, it evaluates how footwear modifications, when paired with AFOs, influence gait biomechanics in both healthy adults, who provide insight into gait adaptations as controlled models, and patients with gait pathology. These findings are intended to inform clinical recommendations and identify directions for future research. A background on ankle-foot biomechanics and the theoretical effects of AFO-FCs in cases of dorsiflexor and plantarflexor weakness is first introduced to provide context for the findings of this review. Existing literature on AFO-footwear interactions in both patient populations and healthy adults is then synthesized to establish an evidence-based overview of the biomechanical outcomes resulting from AFO-footwear interactions.

### Locomotion

Human locomotion can be broken down into 2 main phases, stance and swing. The stance phase is typically defined as the moment the heel of the foot strikes the ground until the foot leaves the ground again [1]. During stance, the ankle-foot complex can be likened to the rolling of a wheel as the center of pressure (COP) moves from heel to toe [15]. Within stance, 3 subphases, known as rockers, can be distinguished as the COP progresses from heel to ankle to forefoot in a curved path relative to a fixed lower-leg coordinate system [16]. The period between initial contact and the forefoot touching the ground makes up the first rocker, also known as the heel rocker. Following this is the second, or ankle, rocker, where dorsiflexion takes place, and the foot remains flat on the ground. The third, forefoot rocker, begins as the heel leaves the ground and concludes with toe-off. The 3 rockers are illustrated in Figure 1.

**Figure 1:**
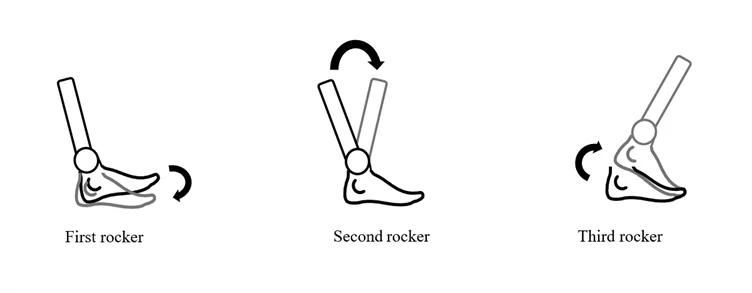
The 3 rockers.

Together, these 3 rockers make up part of a phenomenon known as the “roll-over shape” (ROS). This term refers to the ellipsoidal trajectory of the COP relative to a fixed frame of reference at the ankle [17]. Figure 2 illustrates the transformation from the laboratory reference frame (A) to the ankle-foot reference frame (B). The ROS provides a useful framework for evaluating gait adaptations to AFOs, as it can demonstrate changes in timing and kinematics, providing insight into gait adaptations with a single measure. Figure 3 provides examples of the ROS for healthy individuals (A), two theoretical ROS for patients with calf muscle weakness (B), and visualizations of the ROS when walking with a rigid AFO, both without (C) and with an increased HSD (D). In healthy walking, the ROS is an arc that maintains a relatively constant radius even when walking speed or body weight is altered, due to physiological adaptations of the ankle-foot complex [17,18]. This consistent shape provides a predictable rollover, smoothing step-to-step transitions, allowing humans to walk with minimal energy expenditure [15,19]. Individuals with calf muscle weakness may have a shortened or flattened ROS of the ankle-foot complex compared to the typical arc [20]. Dorsiflexor weakness primarily affects the first rocker, while plantarflexor weakness impairs forward progression of the COP during the second rocker and reduces push-off power during the third rocker [1].

**Figure 2:**
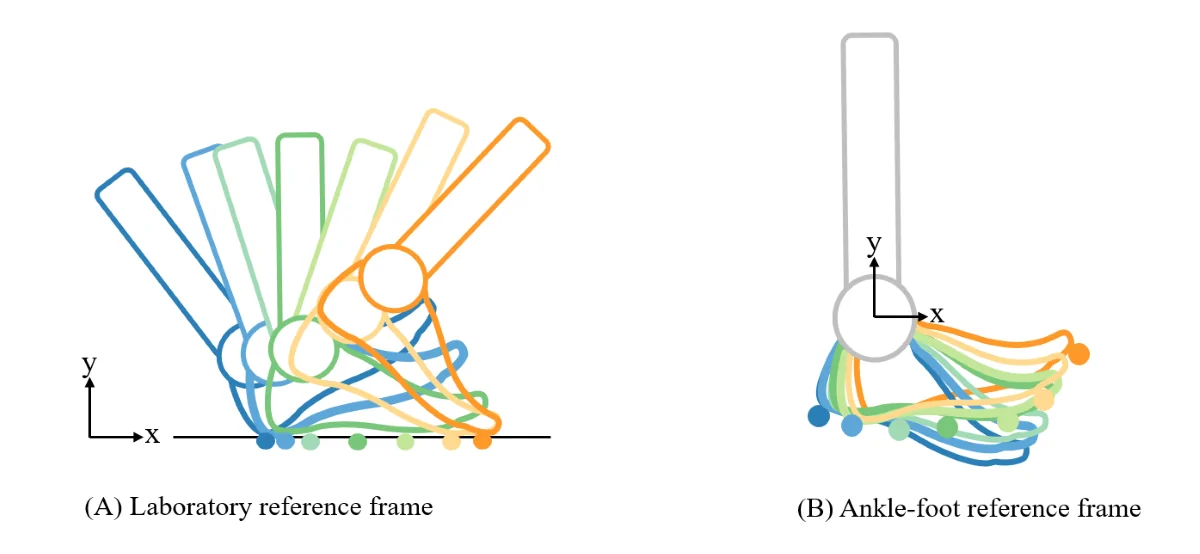
ROS across the 3 rockers. (A) The anteroposterior trajectory of the COP viewed from the global reference frame. (B) The circular trajectory of the COP when the origin is set at the ankle and aligned with the shank, creating the ankle-foot reference frame and forming the roll-over shape. COP: center of pressure; ROS: roll-over shape.

**Figure 3:**
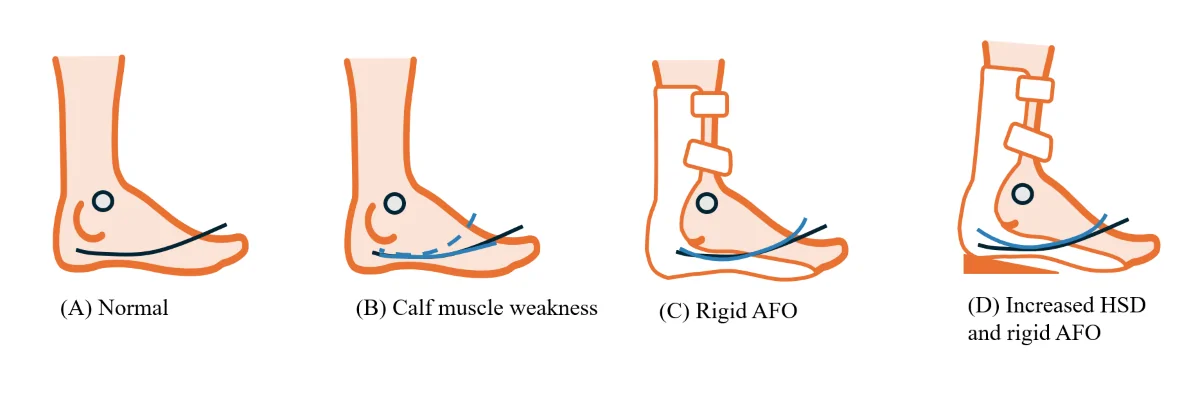
Schematic representation of ROS across clinical and AFO conditions. The gray circle indicates the origin of the ankle-foot reference frame. The black line indicates the normal ROS, and blue lines indicate the ROS under different conditions. (A) ROS for normal healthy walking, based on data from Hansen et al (2004) [18]. (B) Two theoretical ROS for patients with calf muscle weakness. The solid line represents patients with slow COP progression, while the dashed line represents patients with rapid tibial inclination and slow COP progression. (C) ROS when using a rigid AFO, based on data from Fatone et al (2007) [20] and Esposito et al (2021) [16]. (D) ROS when using a rigid AFO with increased HSD, based on data from Esposito et al (2021) [16]. AFO: ankle-foot orthosis; COP: center of pressure; ROS: roll-over shape.

AFOs are commonly prescribed to support patients with calf muscle weakness during gait [3,10,22]. While both passive and active AFOs (exoskeletons) exist, this review focuses on passive AFOs, which are more typically used by patients in daily life. Passive AFOs range from solid polypropylene designs to energy-returning carbon fiber leaf springs and can be tailored to control ankle joint motion to restore a more optimal gait pattern. However, while AFOs can improve stability and help re-establish a smooth ROS [20], their increased stiffness and limited range of plantarflexion motion may reduce push-off power and limit gains in energy efficiency [23].

Footwear plays an important role in gait biomechanics, with design features such as the HSD, rocker soles, and heel hardness influencing how the COP progresses through each rocker relative to the ankle coordinate system [16,24,25]. When combined with AFOs, footwear characteristics can influence orthotic effects. Together, AFO-footwear adaptations offer a powerful means to restore the ROS and improve gait biomechanics in patients with plantar- or dorsiflexor- muscle weakness. However, their effects may differ across the 3 rockers, leading to complex interaction effects between AFO-FCs and gait biomechanics. The following sections summarize the biomechanical effects of the most commonly used AFO-footwear adaptations in the sagittal plane.

### First Rocker

During the first rocker, the dorsiflexors generate a resistive moment that controls plantarflexion, gradually lowering the forefoot to the ground [1]. This negative work from the dorsiflexors leads to smooth knee and hip flexion and supports the controlled transition of the center of mass (COM) from one stance limb to the next [19].

When plantarflexion-restrictive AFOs are used to compensate for dorsiflexor weakness, this gradual transition of the COM is disrupted [26]. The reduced dorsiflexor contribution to the energy transfer between limbs and absence of controlled plantarflexion motion increases angular momentum at the knee and hip. This can result in rapid COP progression toward the midfoot, accompanied by increased knee and hip flexion during early stance [27]. Certain footwear adaptations may mitigate the adverse biomechanical effects of absent plantarflexion during the first rocker. For instance, a shoe with a rocker distal to the heel positions the COP more anteriorly at initial contact [28]. This reduces the ground reaction force (GRF) moment arm at the ankle and knee [28], reducing angular momentum and potentially promoting a smoother COM transition in patients using rigid AFOs. In contrast, a rocker proximal to the heel with a small radius combined with a rigid AFO could theoretically increase the GRF moment arm, thereby increasing angular momentum at the knee and hip.

### Second Rocker

In healthy gait, once the foot is fully in contact with the ground, the gradually increasing plantarflexor activity generates a plantarflexor moment about the ankle joint, decelerating shank inclination and knee flexion [29]. During this phase, near-isometric activation of the plantarflexors combined with ankle dorsiflexion stretches the Achilles tendon, allowing elastic potential energy to be stored and later released as push-off power in terminal stance [29].

Throughout the second rocker, gradually increasing plantarflexor activity shifts the COP toward the forefoot. This progression lengthens the GRF moment arm relative to the ankle joint, contributing to knee and hip extension during terminal stance.

In the case of plantarflexor muscle weakness, insufficient force generation at the ankle may lead to rapid and excessive shank inclination, ankle dorsiflexion, and knee flexion, as well as increased knee and hip extensor moments [1,3]. Excessive knee flexion impairs elastic energy storage in the Achilles tendon and reduces the moment-producing capacities of the ankle plantarflexors, resulting in a flatter ROS and reduced push-off power [20]. A dorsiflexion-resistant AFO can help modulate the rate of forward shank inclination by providing an external plantarflexion moment. This reduces excessive knee and hip flexion, leading to a smoother ROS [3]. Positioning the forefoot apex more distally can prolong the second rocker and extend COP progression [30], potentially increasing external plantarflexion moments and hip and knee extension in AFO users. Conversely, a more proximal apex shortens the second rocker, leading to restricted COP progression and reduced knee extension and plantarflexor moments [12,31].

### Third Rocker

During the third rocker, the heel lifts off the ground and COP progression and ankle plantarflexor moments approach their peak. At this stage, body weight rapidly transitions from single-limb to double-limb support and elastic energy stored in the Achilles tendon is rapidly released, generating positive ankle power and facilitating a smooth redirection of the COM into the subsequent stance phase, as well as rapid swing initiation of the trailing limb [1]. Adequate timing and sufficient magnitude of peak ankle power ensure rapid rotation of the shank-foot system over the forefoot [19].

The increase in plantarflexion moment during the second rocker contributes to forward progression of the COP and a gradual increase in ankle angular velocities [1]. As the COP progresses anteriorly toward the forefoot, it approaches the shoe’s forefoot apex, which effectively acts as a rotational axis for the foot-shoe system. When the COP reaches this apex position, the external dorsiflexion moment generated by the GRF can no longer balance the internally generated plantarflexion moment, resulting in rotation of the foot about the forefoot apex and heel lift-off, marking the onset of the third rocker.

AFOs with energy-storing elements (such as springs or carbon fiber elements) can store mechanical energy during the second rocker and release it at push-off (third rocker) to aid propulsion. A more distal apex position could delay the onset of the third rocker, increasing COP progression, peak GRF moment arms, and ankle dorsiflexion, leading to greater energy storage in spring-like AFOs and enhanced push-off power when the energy is released in the third rocker. Additionally, changes in rocker radius can modulate timing and duration of foot-to-ground contact and shank kinematics during the third rocker, where a smaller radius accelerates shank inclination and energy release (smaller ROS arc), while a larger radius decelerates it, prolonging the third rocker phase (larger ROS arc). Table 1 summarizes the theoretical biomechanical effects across all 3 rockers for 3 types of footwear adaptations when used with an AFO that restricts both plantar- and dorsiflexion.

**Table 1:** Overview of theoretical kinematic and kinetic outcomes for different footwear adaptations with the use of an AFO^a^ restricting plantarflexion and dorsiflexion.

			Heel-sole differential		Heel rocker apex position, radius/angle		Forefoot rocker apex position, radius/angle
**Kinematics **							
	First rocker	Increasing HSD^b^ increases SVA^c^ and knee flexion		Larger radius/smaller rocker angle decreases rate of forefoot lowering		N/A^d^	
	Second rocker	Increasing HSD increases SVA and knee flexion		N/A		N/A	
	Third rocker	N/A		N/A		Smaller radius/larger rocker angle increases SVA and knee flexion and increases angular velocity of the foot-ankle-shank complex	
**Kinetics**							
	First rocker	N/A		More distal heel AP^e,f^ reduces ankle DF^g^ moment Smaller radius increases ankle DF moment		N/A	
	Second rocker	Increasing HSD increases knee extensor moment		N/A		N/A	
	Third rocker	N/A		N/A		Smaller radius/larger rocker angle decreases peak knee extensor moment More distal forefoot position increases PF^h^ moment	

^a^AFO: ankle-foot orthosis.

^b^HSD: heel-sole differential.

^c^SVA: shank-to-vertical angle.

^d^N/A: not applicable.

^e^AP: apex position

^f^For the purpose of this review, a more proximal heel radius refers to an apex position proximal to the heel.

^g^DF: dorsiflexion.

^h^PF: plantarflexion.

## Methods

### Study Design

This systematic review was registered with PROSPERO (International Prospective Register of Systematic Reviews; CRD42024625819) and is reported in line with the PRISMA (Preferred Reporting Items for Systematic Reviews and Meta-Analyses) guideline [32].

### Search Strategy

A systematic database search was performed by the primary reviewer using PubMed, Embase, Scopus, and Web of Knowledge. The search was performed using the following phrases: ankle foot ortho*, AFO, AFO-FC, orthotic*, orthosis, orthoses, splint, combined with biomech*, gait, locomotion, walk*, kinemat*, and adaptation, shoe, footwear, rocker, roll-over, insert, wedge, and tuning. Papers were retrieved from the beginning of the respective database history until December 2024. A follow-up search was conducted in May 2026. The complete search string for Scopus is:

TITLE-ABS-KEY((“ankle foot ortho*” OR “orthotic device” OR AFO OR “AFO-FC” OR (ankle AND (orthotic* OR orthosis OR orthoses OR splint))) AND

(biomech* OR gait OR “gait analys*” OR locomotion OR walk* OR kinemat* OR tuning OR manipulation) AND (shoe* OR footwear OR rocker* OR “roll-over” OR insert* OR wedge* OR tuning))

The remaining search strings, including database-specific keywords, can be found in Multimedia Appendix 1.

### Eligibility Criteria

Titles were excluded if they mentioned nonadult participants, animal participants, or cadavers. Review papers, protocols, or papers that investigated only movements other than walking, surgical techniques or surgery-related outcomes, or devices that were not AFOs, lower leg casts, or footwear were also excluded in the title exclusion process. Rigid lower leg (plaster) casts were considered to behave in a similar manner to rigid AFOs and were eligible for inclusion.

Abstracts were excluded if the abstract did not mention both footwear and AFOs and did not describe effects of footwear adaptation on gait while using an AFO. The abstract must have described walking with an AFO in at least two footwear conditions. Abstracts were excluded when footwear was manipulated in only the leg contralateral to the AFO.

Papers written in English, French, German, or Dutch were eligible for full-text selection. Inclusion criteria required that the study described or visualized the AFOs, footwear, and footwear modifications. To qualify, the footwear adaptation must have been applied externally to the AFO, either between the AFO and the shoe, the shoe itself, or between the shoe and the ground. Additionally, at least one of the following outcome measures must have been examined: sagittal plane lower-limb joint kinetics and kinematics, sagittal plane whole-body COM kinetics and kinematics, spatiotemporal walking parameters, muscle-tendon dynamics, lower-limb EMG (electromyography), COP, GRF, plantar pressure, or metabolic cost.

### Selection Process

Screening was performed with the use of Catchii, a systematic review management tool [33]. Two reviewers independently screened papers at each stage of the inclusion process. Reviewers AM and CG performed the title and full-text screening, and the abstract inclusion process was performed by AM and JMH. Disagreements were resolved through discussion between each pair of reviewers. For each stage of the selection process, papers were excluded based on the first exclusion criterion that was met, and only that criterion was recorded.

### Quality Assessment and Data Extraction

The quality of the included research was assessed using a modified Downs and Black Checklist [34]. This version of the checklist scored question 27 binarily, rather than out of the original 5 points, and is more suited to observational studies. Questions regarding follow-up were omitted from the checklist as gait studies are typically within-participant designs without follow-up. The final checklist was scored out of a potential 25 points. Adjusting for the omitted questions, a study could be considered “excellent” quality if it scored 23-25 points, “good” between 17 and 22 points, “fair” between 12 and 16 points, and “poor” if marked less than 12 points [35]. Only studies scoring “fair” or higher would be considered to have sufficient quality for data extraction.

A modified version of the AFO reporting checklist developed by Ridgewell et al [36] was used to evaluate the quality of AFO reporting. The checklist evaluates 3 categories: participant details, AFO details, and the experimental protocol. This checklist was developed for studies on children with cerebral palsy, and as such the section on participant details was excluded for the purpose of this review. Scoring followed the guideline provided by Ridgewell et al [36]. Reviewers AM and CG independently scored both checklists. Any disagreements were resolved through discussion.

Data were extracted by AM. A custom checklist was used to extract footwear design parameters. This checklist includes design descriptors such as outsole hardness and apex position but does not include manufacturing items such as last type or sole material. Only outcome measures listed in the inclusion criteria were included in this review. Data were systematically synthesized using a structured biomechanically informed framework. Data were synthesized first by footwear adaptation (eg, HSD), then by the phase of gait in which outcomes were measured, and finally by the specific outcome measure. For clarity purposes, all moments are reported as internal moments, with any values reported as external moments transformed by AM.

## Results

### Search Results

The systematic database search, including the updated search, resulted in 3064 papers after duplicates were removed. A total of 118 full texts were evaluated for inclusion in the review. Of these, 9 papers were written in German, and 1 paper in French, all of which were excluded due to not being primary research. A total of 14 studies were included in the review, following the inclusion procedure outlined in Figure 4.

**Figure 4:**
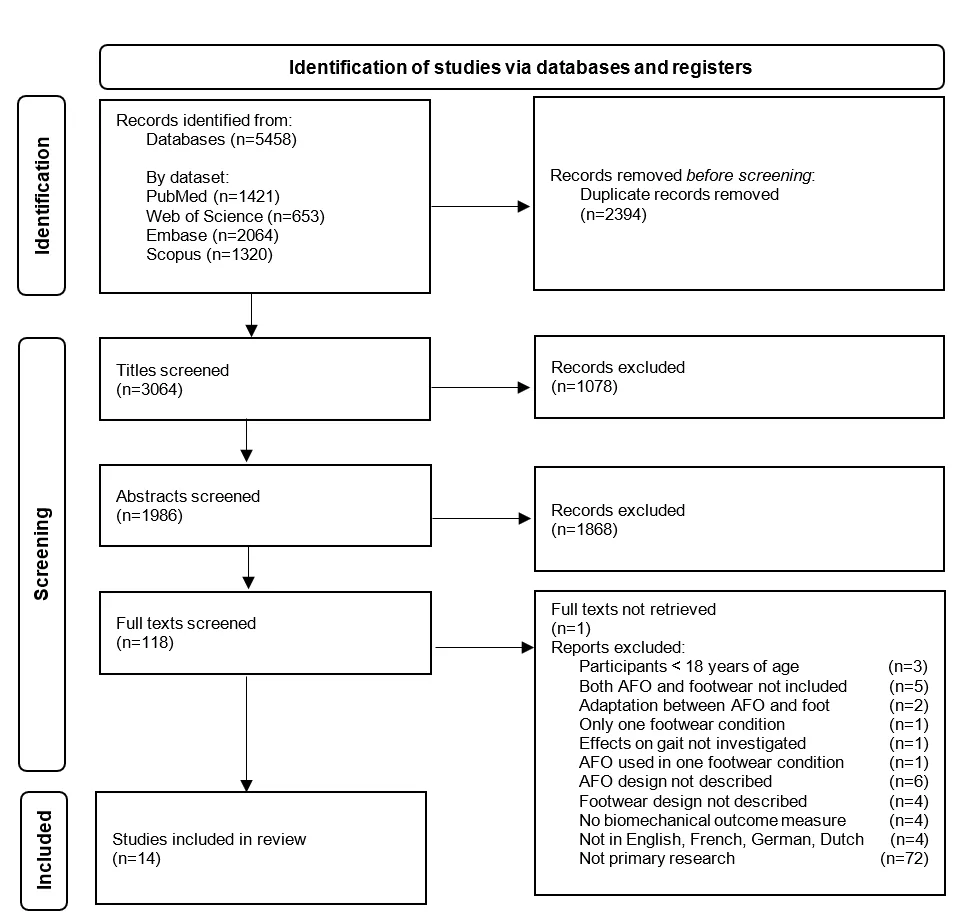
Study selection flowchart including updated search. Exclusion criteria shown here were those used to exclude full texts. AFO: ankle-foot orthosis.

### Study Characteristics

An overview of the participants, interventions, and study designs can be found in Table 2. The majority (10/14) of studies were within-subjects designs evaluating hemiparetic patients after stroke using solid dorsal AFOs.

**Table 2:** Summary of included studies.

Author, year	Participant characteristics	Pathology	Study design	Gait speed^a^	Type of AFO^b^	Footwear modification	Quality score (0-25)
Wiest et al [28], 1979	9 adults, aged 19-46 years	None	Prospective within-subjects	Outcome	Bichannel adjustable AFO	Heel hardness and heel shape	17
Farmani et al [37], 2016	18 adults aged 57.86 ± 10.44 years	Hemiplegia secondary to stroke	Prospective within-subjects	Outcome	Custom rigid AFO	Rocker outsole attached directly to AFO footplate	19
Sutdet et al [38], 2019	4 adults aged 51 ± 15.2 years	Right-sided hemiplegia and genu recurvatum secondary to stroke	within-subjects pilot	Outcome	Custom rigid AFO	Insole wedge height, location unspecified	18
Dhalla et al [39], 2003	28 adults, age not given	None	Prospective within-subjects	NS^c^	Custom fabricated total contact cast	Conventional cast shoe, rigid rocker cast shoe, flat rubber heel attached directly to cast, rubber rocker heel attached directly to cast	16
Farmani et al [40], 2016	30 adults, aged 49-68 years, mean weight 64.7 kg	Hemiplegia secondary to stroke	Prospective block randomized	Outcome	Prefabricated solid polypropylene AFO	High-top rocker shoes, high-top flat-soled shoes	19
Kearney et al [41], 2011	15 adults aged 31.3 ± 4.7 years, height 172 ± 9 cm, and weight 70 ± 15 kg	None	Prospective within-subjects	Self-selected per condition	Rocker bottom walking cast and 2 different carbon-fiber AFOs	Insole heel wedge height	18
Arvin et al [42], 2013	24 adults aged 24.7 ± 4.84 years	None	Prospective within-subjects	Outcome	Modified solid AFO with capability of locking at 5°-7° dorsiflexion	Standard shoe and AFO compared to AFO with inclined shank without a shoe	18
Jagadamma et al [43], 2010	Female person aged 61 years	Left side hemiplegia secondary to stroke	Case study	Outcome	Custom solid polypropylene AFO	External heel wedge height with and without a stiff rocker outsole	17
Masoumi et al [44], 2020	10 adults aged 53.6 ± 7.5 years, height 167.1 ± 5.93 cm, weight 71.3 ± 8.53 kg	Hemiplegia secondary to stroke	Prospective within-subjects	Outcome	Custom rigid AFO	Rocker shoe	19
Kerkum et al [45], 2015	10 adults, aged 24 ± 3 years	None	Prospective within-subjects	Self-selected^d^	Prefabricated carbon AFO	Insole heel wedge height, AFO footplate stiffness	19
Ikeda et al [46], 2018	24 adults aged 18-55 years	Unilateral foot or ankle weakness or pain; controls with no pathologies	Prospective repeated-measures crossover design	Nonself-selected^d^	Carbon fiber AFO	Insole heel wedge height and hardness	16
Choi et al [47], 2016	Male person aged 56 years, height 190 cm, weight 88.5 kg	Left-sided hemiplegia secondary to stroke	Case study and model	Outcome	Prefabricated posterior leaf spring AFO; solid polypropylene AFO	Shoe heel height	14
Daryabor et al [48], 2021	20 adults aged 33-65 years, height 1.62 ± 0.1 / 1.6 ± 0.1 m, weight 67.8 ± 8.7 / 67.1 ± 10.5 kg	Hemiplegia secondary to stroke	Randomized controlled trial	Outcome	Custom PF^e^-stop AFO; custom PF-resistant AFO	Rocker shoe	16
Schmalz et al [49], 2006	10 adults aged 34 ± 9 years, height 178 ± 4 cm, weight 73 ± 9 kg	None	Prospective within-subjects	Self-selected per condition	Rigid AFO; semirigid supramalleolar orthosis	Medial and lateral wedges external to shoe	17

^a^The gait speed column indicates whether gait speed was an outcome variable or controlled across conditions.

^b^AFO: ankle-foot orthosis.

^c^NS: not stated.

^d^Denotes the gait speed was controlled across conditions.

^e^PF: plantarflexion.

### Participant Characteristics

A total of 204 participants (108 healthy, 84 patients after stroke, and 12 with unilateral ankle weakness or pain) were included across all 14 studies. Seven papers examined AFO-footwear combinations in patients with hemiparesis secondary to stroke [37,38,40,43,44,47,48]. Hemiparetic participants ranged across studies from 11 months to over 10 years post stroke onset. Six studies recruited only healthy participants [28,39,41,42,45,49], and one study enrolled both healthy controls and participants with unilateral foot or ankle weakness or pain [46]. Of the 8 studies that included patients, 5 studies recruited participants who used AFOs in their daily lives [37,38,43,46,47], 1 study included only participants who had not used AFOs in their daily lives [48], and 2 studies did not specify whether AFOs were used in daily life [40,44]. Nine studies included gait speed as an outcome measure [28,37,38,40,42-44,47,48]. Three studies let participants walk at a self-selected walking speed and did not report it as an outcome. Of these 3 studies, one study controlled the speed between the trials [45], and 2 studies did not specify whether speed was controlled between conditions [41,49]. One study set a target gait speed based on the participants' leg length and a Froude number of 0.16 and used this for all conditions [46]. One study did not report gait speed as an outcome or specify if gait speed was controlled [39].

### AFO Properties

A summary of the AFOs included in this review and their properties can be found in Table 3. A total of 19 different AFOs were used across the 14 included studies. Eight of the AFOs used a solid dorsal shell design constructed out of polypropylene [37,38,40,42-44,47]. One study used 2 AFOs with a “plastic shell,” which are assumed to be polypropylene dorsal shell AFOs, one with plantarflexor resistance and one with a plantarflexion stop [48]. Four AFOs were carbon-fiber, of which 2 AFOs had ventral shells [41], 1 AFO a proximal cuff [46], and 1 AFO a dorsal shell [45]. While a walking boot was also investigated by Kearney et al [41], results from the cast were not included, as it did not meet the inclusion criteria of having the footwear adaptation be between the AFO and the shoe or ground. One AFO had a hinged ankle joint with adjustable medial and lateral struts which attached directly to an experimental shoe [28]. One study used 2 AFOs with the same design with either a medial or lateral strut [49]. These 2 AFOs were counted as 1 AFO in Table 3 as they were otherwise identical. The AFO had free dorsiflexion and plantarflexion but did not permit frontal plane motion. No information about any parameters other than strut position was given or possible to infer for this AFO. This study also examined a stirrup brace which restricted frontal plane movement and was considered an AFO. A plaster cast [39] was reported and categorized as an AFO for the purpose of this review. Including the cast, 11 AFOs had full-length footplates [37,39-42,44,46,47]. The length of the footplate was not stated or possible to infer from images for 7 AFOs [38,43,45,47-49]. Eight of the footplates were rigid [37-39,42,44,45,47], one was flexible throughout the footplate [45], and one was flexible at the metatarsophalangeal joint [43]. Two AFOs had indeterminable footplate stiffnesses, with the only description given as one AFO being “more flexible,” and the other AFO being “less flexible” [41]. The footplate stiffness was not given or impossible to infer for 7 AFOs [28,40,47-49].

**Table 3:** Overview of AFOsa.

Author, year	Design	Materials	Movement restrictions	Ankle joint (rigid, leaf spring, stop)	Ankle angle^b^ (°)	Footplate length	Footplate stiffness
Wiest et al [28], 1979	Custom^c^ instrumented bichannel with adjustable height and locking ankle joint	NS^d^	DF^e^ and PF^f^	Locked hinge	90	Directly attached to shoe	NS
Farmani et al [37], 2016	Custom solid dorsal	3-mm thick polypropylene	DF and PF	Rigid	90	Full length (heel to tip of toes)	Rigid (inferred)
Sutdet et al [38], 2019	Custom solid dorsal	5-mm thick polypropylene	All directions	Rigid, reinforced with 3-mm polypropylene	90	NS	Rigid (inferred)
Dhalla et al [39], 2003	Custom total contact cast	Orthoflex plaster with fiberglass shell	All directions	Rigid	90	Full length (heel to slightly beyond tip of toes)	Rigid
Farmani et al [40], 2016	Prefab^g^ solid dorsal	Polypropylene	NS	Rigid	90	Full length	NS
Kearney et al [41], 2011	Prefab ventral	Carbon-fiber	NS	Leaf spring	90	Full length	“Less flexible”
Kearney et al [41], 2011	Prefab ventral	Carbon-fiber	NS	Leaf spring	90	Full length	“More flexible”
Arvin et al [42], 2013	Custom solid dorsal	4-mm thick polypropylene	All directions	Rigid	90	Full length	Rigid (inferred)
Arvin et al [42], 2013	Custom solid dorsal	4-mm thick polypropylene	All directions	Rigid	83-84	Full length	Rigid (inferred)
Jagadamma et al [43], 2010	Custom solid dorsal	4-mm thick polypropylene	NS	Rigid	90	NS	Flexible at MTP^h^ joints
Masoumi et al [44], 2020	Custom solid dorsal	4-mm thick polypropylene	NS	Rigid	90	Full length	Rigid (inferred)
Kerkum et al [45], 2015	Prefab solid dorsal	Prepreg carbon	All directions	Rigid (7.9 Nm/deg)	90	NS	0.06 Nm/deg and 0.89 Nm/deg + 0.06 Nm/deg
Ikeda et al [46], 2018	Custom proximal cuff	Carbon-fiber	PF	Flexible strut	NS	Full length	Rigid
Choi et al [47], 2016	Prefab posterior leaf spring	NS	NS	Leaf spring	NS	NS	NS
Choi et al [47], 2016	Custom solid dorsal	Polypropylene	NS	Rigid	82	Full length	NS
Daryabor et al [48], 2021	Custom, dorsal	Plastic	PF	PF stop	90	NS	NS
Daryabor et al [48], 2021	Custom, dorsal	Plastic	PF	PF resistive spring	90	NS	NS
Schmalz et al [49], 2006	Prefab stirrup ankle brace	Textile and plastic	Mediolateral	Flexible	N/A^i^	N/A	N/A
Schmalz et al [49], 2006	Customization unknown, medial/lateral strut	NS	Mediolateral	NS	NS	NS	NS

^a^AFO: ankle-foot orthosis.

^b^Ankle angle is taken as the angle between the footplate and the shank of the ankle-foot orthosis.

^c^All participants used the same ankle-foot orthosis, which was a custom-made experimental ankle-foot orthosis that could be adjusted to each participant.

^d^NS: not stated.

^e^DF: dorsiflexion.

^f^PF: plantarflexion.

^g^Prefab: prefabricated.

^h^MTP: metatarsophalangeal.

^i^N/A: not applicable.

### Footwear Adaptations

The footwear adaptations used for each study can be found in Table 4. Footwear adaptations are reported in the same manner as the respective study. Seven papers investigated adaptations at the heel of the shoe. The majority of these focused on increasing the HSD, either with insole wedges [41,45,46] or through altering the heel height of the shoe itself [47]. An overview of these adaptations can be examined in Table 5. One study used insoles to manipulate the SVA, but did not specify the shape, height, or location of these insoles [38]. One paper examined the effects of changing the shape and hardness of the heel of an experimental shoe [28]. One paper examined the effects of increasing the HSD using wedges, which were then translated to the design of a custom shoe and combined with a stiff rocker outsole [43]. Design parameters for that rocker outsole were not given; however, from the image provided, it can be inferred that an apex position proximal to the metatarsophalangeal joint was likely used.

**Table 4:** Overview of footwear adaptations with respect to the shoe profile. Comparison condition refers to the condition with the AFOa without any footwear adaptations and may not be the control condition. Footwear adaptations follow the naming conventions used per respective study.

Author, year	Comparison condition	Footwear adaptation	Forefoot/heel rocker	Rocker angle/radius	Apex position	Heel sole hardness	Outsole hardness	Midsole hardness and distribution
Wiest et al [28], 1979	Dress shoe with hard (Shore-A 74) rubber heel and AFO	SACH^b^	None	N/A^c^	N/A	Medium density polyurethane center	NS^d^	NS
Wiest et al [28], 1979	Dress shoe with hard (Shore-A 74) rubber heel and AFO	Heel rocker	Heel	20°	3.5 cm from distal edge of heel	Shore-A 74	NS	NS
Wiest et al [28], 1979	Dress shoe with hard (Shore-A 74) rubber heel and AFO	Compressible heel	None	N/A	N/A	Shore-A 40	NS	NS
Farmani et al [37], 2016	AFO with thin nonslip rubber outsole attached directly to AFO footplate	Rocker sole applied directly to AFO (no shoe)	FF^e^	15°	65%	N/A	Shore-A 50-60	Shore-A 30-40, length of footplate
Sutdet et al [38], 2019	Sport shoe with AFO without wedges	Increasing HSD^f^	None	N/A	N/A	NS	NS	NS
Dhalla et al [39], 2003	Cast with no shoe	Conventional cast shoe	FF and heel	NS	NS	N/A	NS	NS
Dhalla et al [39], 2003	Cast with no shoe	Rigid rocker bottom shoe	FF and heel	NS	NS	N/A	“Rigid”	N/A
Dhalla et al [39], 2003	Cast with no shoe	Rubber rocker bottom applied directly to cast, centered on longitudinal arch of the foot	Heel	NS	NS	NS	NS	N/A
Dhalla et al [39], 2003	Cast with no shoe	Flat rubber heel applied directly to cast in line with the tibia	N/A	N/A	N/A	N/A	NS	N/A
Farmani et al [40], 2016	High-top standard shoe with AFO	Rocker sole applied to standard shoe	FF	15°	65%	NS	NS	Shore-A 30-40, length of shoe
Kearney et al [41], 2011	Participant’s own shoe with AFO; without wedges	Increasing HSD	None	NS	NS	NS	NS	NS
Arvin et al [42], 2013	Sport shoe with 1 cm HSD and AFO	AFO with inclined shank (5°-7° anterior inclination)	None	N/A	N/A	N/A	N/A	N/A
Jagadamma et al [43], 2010	Standard shoe with AFO	Rocker sole applied to standard shoe and changes to HSD	FF	NS	NS	NS	Rigid	NS
Masoumi et al [44], 2020	Standard shoe with AFO	Rocker sole applied to standard shoe	FF	15°	65%	NS	Shore-A 50-60	Shore-A 30-40, length of shoe
Kerkum et al [45], 2015	Flexible flat-soled shoe (Converse-style sneakers)	Increasing HSD	None	N/A	N/A	NS	NS	NS
Ikeda et al [46], 2018	Sports shoe and AFO without wedges	Increasing HSD	None	N/A	N/A	NS	NS	NS
Choi et al [47], 2016	Sports shoe and AFO without wedge	Material removed from heel of shoe	None	N/A	N/A	NS	NS	NS
Daryabor et al [48], 2021	Standard shoe with AFO	Rocker sole applied to standard shoe	FF	15°	65%	NS	Shore-A 50-60	Shore-A 30-40, length of shoe
Schmalz et al [49], 2006	Dress shoe and AFO without wedge	Mediolateral differential	None	N/A	N/A	NS	NS	NS

^a^AFO: ankle-foot orthosis.

^b^SACH: solid ankle cushion heel

^c^N/A: not applicable.

^d^NS: not stated.

^e^FF: forefoot.

^f^HSD: heel-sole differential.

**Table 5:** Overview of heel-sole differential adaptations.

Author, year	Wedge used^a^ (Y^b^/N^c^)	Insole or external	Location	Shape	Thickness (cm)	Hardness
Sutdet et al [38], 2019	Y	Insole	NS^d^	NS	Inferred from SVA^e^	NS
Kearney et al [41], 2011	Y	NS	Heel	NS	1.5, 3.0, 4.5	NS
Jagadamma et al [43], 2010	Y	NS	Heel	NS	Inferred from SVA	NS
Kerkum et al [45], 2015	Y	Insole	Heel	NS	0.6/1.3; 2.8/2.8; 4.9/5.3 cm	NS
Ikeda et al [46], 2018	Y	Insole	Heel	NS	1.0, 2.0, 3.0	Per each thickness: 50-57, 75-78 on OO^f^ scale
Choi et al [47], 2016	N	External	Heel	NS	NS	N/A^g^
Schmalz et al [49], 2006	Y	External	Medial/lateral; highest point at heel of shoe	Tapered 14° angle of horizontal inclination	10 mm (at highest point)	Firm

^a^“Wedge used” refers to whether a separate piece of material was used to increase the heel-sole differential.

^b^Y: yes.

^c^N: no.

^d^NS: not stated.

^e^SVA: shank-to-vertical angle.

^f^OO: shore OO hardness.

^g^N/A: not applicable.

Three studies compared standard shoes to rocker shoes [40,44,48]. All 3 studies used the same forefoot rocker design of an apex position at approximately 65% of the length of the shoe from the heel, and a rocker angle of 15° with a curved forefoot and stiff outsole. Farmani et al (2016) [37] studied the effects of adding a rocker sole directly to the footplate of a rigid polypropylene AFO compared to the same AFO with only a layer of nonslip rubber added to the footplate [37]. This rocker sole also had an apex position at approximately 65% of the shoe length with a beveled forefoot with a rocker angle of 15°, and as such is considered functionally a rocker shoe for the purpose of this review.

Dhalla et al [39] examined the effects of different types of cast shoes when used with a rigid walking cast. Schmalz et al [49] examined the effects of mediolateral wedging, in this instance applying the wedges externally to the shoe. It is unclear whether the footplate of the AFO and wedge were the same length. A study from Arvin et al [42] compared a conventional solid dorsal AFO with a shoe to an AFO of the same manufacturer but with a shank inclined in dorsiflexion without a shoe. Both the traditional AFO with a shoe and the modified AFO worn without a shoe had the same SVA of 5°-7° inclination [42].

### Quality Assessment

#### Study Quality

The scores for each included paper can be found in Table 2. The score each paper received per question can be examined in Multimedia Appendix 2 [28,37-49]. The majority of papers (10/14) were considered “good” quality, with the remaining papers (4/14) considered “fair.” The lowest score given was 14. No paper received a “yes” for stating whether an attempt was made to blind either the participants or those measuring the outcomes.

#### AFO Reporting

Multimedia Appendix 2 provides an overview of the results of the AFO Reporting Checklist. A total of 19 different AFOs were used across the 14 included studies, with 2 studies using 2 different AFO designs, and 1 study comparing a walking boot to 2 different AFOs. The majority (10/14) of studies applied their modifications in a randomized order, 2 studies did not state whether randomization occurred, and 2 studies were case studies where randomization was not applicable. Over half (12/19) of the AFOs were custom manufactured, with only 1 AFO’s manufacturing status being unclear. A clear motivation for why a specific AFO design was used was given for all AFOs. No consistent method of reporting AFO details was present across the included studies. AFO design details often had to be inferred from photos, with 8 of the AFOs having completely clear trimlines. The stiffness and ankle position were described in detail for 1 AFO. A clear description of the movements assisted or prevented was given for 13 of the AFOs.

#### Biomechanical Effects

Table 6 provides an overview of the outcome measures extracted for this review. Only outcome measures fitting the inclusion criteria outlined above were included for the purpose of this review. These outcome measures can be divided into 5 main categories: kinematics, kinetics, plantar pressure, spatiotemporal parameters, and GRF. Table 7 provides the effects of each type of footwear adaptation per outcome measure across the 3 rockers. All results are given for rigid AFOs unless otherwise specified.

**Table 6:** Outcome measures of interest.

Outcome measure			Included from reference number		Total patients (n)		Total healthy (n)
**Kinematics**							
	Ankle	[44^a^,^46^-^48^]		1 S^b^, 12 U^c^		20	
	Knee	[27,36^a^,^42^,^44^,^46^-^48^]		40 S		29	
	Hip	[36^a^,^44^,^46^,^47^]		39 S		10	
**Kinetics**							
	Ankle	[45-49]		45 S		20	
	Knee	[28,38,43,45-49]		26 S, 12 U		29	
	Hip	[45,47,48]		21 S		10	
Plantar pressure			[39,41]		0		43
Spatio-temporal			[27,36^a^,^37^,^39^-^43^^a^,^45^-^48^]		84 S, 12 U		58
GRF^d^			[28,48,49]		20 S		19
Other			[45,46]		12 U		10

^a^Denotes a significant difference between the standard shoe with AFO and at least one adapted shoe with AFO condition. Statistically significant results comparing a non-AFO condition to adapted shoe conditions are not indicated.

^b^S: patients after stroke.

^c^U: patients with unilateral weakness or pain.

^d^GRF: ground reaction force.

**Table 7:** Overview of outcomes across the gait cycle for different footwear adaptations when used with a rigid AFOa.b

Heel-sole differential			Heel rocker		Heel hardness		Forefoot rocker
**Kinematics**							
	**First rocker**						
		Increasing HSD^c^ increases knee flexion [43] (n=18S)^d^	Use of heel rocker decreases knee flexion [28] (n=9H)	More compressible heel decreases knee flexion [28] (n=9H)		N/A^e^	
	**Second rocker**						
		Increasing HSD increases knee flexion [38] (n=4S), [43] (n=1S), [45] (n=10H) and hip flexion [45]^d^	N/A	N/A		N/A	
	**Third rocker**						
		Increasing HSD increases knee flexion [43] (n=18S), [47] (n=1S)	N/A	N/A		Increases knee flexion at toe-off [37] (n=18S)d Earlier knee flexion during preswing [43] (n=1S)	
**Kinetics**							
	**First rocker**						
		Increasing HSD increases knee extensor moment [46] (n=12U)^d^	Decreases knee extensor moment [28] (n=9H)^d^	More compressible heel decreases ankle DF^f^ [46] (n=12U) and knee extensor moments [28] (n=9H)		N/A	
	**Second rocker**						
		Increasing HSD increases knee extensor moment [47] (n=1S)^d^	N/A	N/A		N/A	
	**Third rocker**						
		N/A	N/A	N/A		GRF^g^ moved posterior to the knee joint during terminal stance and preswing [43] (n=1S)	
**Spatio-temporal**							
		No effect on walking speed [38] (n=4S)	No effect on cadence or walking speed [28] (n=9H)	—^h^		Increases walking speed with rigid AFO [37] (n=18S), [44] (n=10S)	
**Plantar pressure**							
		—	Decreases peak hindfoot plantar pressure [39] (n=28H)	—		Decreases peak forefoot plantar pressure [39] (n=28H)	

^a^AFO: ankle-foot orthosis.

^b^The number of participants is given in brackets beside the respective reference. Whether the participants are healthy (H), patients after stroke (S), or have unilateral weakness or pain (U) is indicated.

^c^HSD: heel-sole differential.

^d^Effects are in line with the theory presented in Table 1.

^e^N/A: not applicable.

^f^DF: dorsiflexion.

^g^GRF: ground reaction force.

^h^Not available.

#### HSD

Increasing the HSD showed effects on gait across all 3 rocker phases, with insole wedges being used to influence biomechanics in the third rocker [43]. Increasing the HSD from 1 to 3 cm increases the peak dorsiflexor moment at the beginning of the second rocker, shifting the second rocker later in stance [46]. This change in HSD also increases the time required for the COP to reach peak anteroposterior velocity. Peak COP velocity occurred approximately 10% later in the stance phase when using a soft 3 cm wedge compared to a soft 1 cm wedge, and just over 30% of the stance phase later for a firm 3 cm wedge compared to a firm 1 cm wedge [46]. The peak knee extensor moment during loading response increases and occurs later in the gait cycle as the HSD increases [46].

An increase in HSD increases the SVA in both healthy individuals and patients after stroke, resulting in greater knee flexion [38,43,45]. Similarly, a larger HSD increases the ankle dorsiflexion angle, plantarflexor moment, and hip flexion angle at midstance [45].

In the third rocker, the use of an increased HSD and rigid AFO elicited increases in knee flexion at toe-off when compared to a posterior leaf spring–AFO and standard shoes in a patient who had a stroke [47]. Larger plantarflexor and knee extensor moments in a patient who had a stroke were also observed at toe-off when compared to walking in only unmodified shoes [47].

Increasing the HSD linearly increased the radius of curvature of the ROS normalized to participant body height, with 3 cm wedges producing a radius of curvature in 4% of body height greater than 1 cm wedges [46].

A linear increase in forefoot pressure and simultaneous decrease in hindfoot pressure was found with increasing HSD [41]. There was no difference found between AFO stiffnesses when the same changes in HSD were applied [41].

Increasing the HSD did not affect walking speed but did decrease cadence in patients after stroke when compared to walking with shoes only, with a significant decrease in cadence when the shank was inclined by 10° [38]. There was also no difference in the duration of terminal stance regardless of AFO stiffness or HSD [41]. Similarly, there were no significant differences for speed, step length, or step width in healthy participants when the SVA is held constant between an AFO with an inclined shank and no shoe, and an AFO with a neutral SVA and a shoe with a 1 cm HSD [42].

#### Heel Rocker

Three different heel rockers were investigated across 2 studies. A heel with a 20-degree bevel reduced the knee extensor moment during loading response by 24% compared to a conventional men’s dress shoe hard rubber heel when both were used with a rigid AFO [28]. The addition of a heel rocker to a walking cast allowed for a reduction in peak hindfoot pressure [39]. Conversely, a rigid whole-foot rocker applied to the same cast did not decrease hindfoot pressure; however, it did decrease peak forefoot plantar pressure [39].

#### Heel Hardness

Increasing the hardness of the heel external to the shoe increased the knee extensor moment during the loading response, with a hard rubber heel having a moment 31% higher than a SACH (solid ankle cushion heel), and 12% higher than a compressible crepe heel [28]. Harder heel wedges increased the peak ankle dorsiflexor and knee extensor moments during second rocker [46]. Increasing both the height and hardness of a heel wedge significantly delayed the timing during the stance phase of peak COP velocity, with the peak occurring approximately 15% later in stance for a firm 3 cm wedge compared to a compressible 1 cm wedge [46].

#### Forefoot Rocker

The use of a proximal forefoot rocker increased knee flexion at toe-off when used with a rigid AFO [37,43]. This increase in knee flexion also resulted in the GRF being more posterior to the knee joint during third rocker compared to a shoe with a standard forefoot [43]. In both plantarflexion stop and plantarflexion resistant AFOs, the addition of a proximal forefoot rocker significantly reduced the peak ankle plantarflexor moment when compared to the same AFOs and standard shoes [48].

In rigid AFOs, the addition of a proximal forefoot rocker increased walking speed when compared to the same AFO with a standard shoe [40,44]. Conversely, the use of a proximal forefoot rocker with dorsiflexion-free AFOs resulted in slower walking speeds when compared to those same AFOs and a standard shoe [48]. The stride length was also reduced in patients walking with dorsiflexion-free AFOs when paired with a proximal forefoot rocker compared to when paired with a standard shoe.

#### Mediolateral Wedging

Mediolateral wedging had limited effects on sagittal plane biomechanics. The peak knee extensor moment during third rocker was reduced with the use of either a medial or lateral wedge, although only significantly with the medial wedge, when paired with an AFO that restricted frontal plane motion at the ankle joint [49]. Medial or lateral wedging with either an ankle support or rigid AFO decreased the vertical component of the GRF when compared to a shoe without any wedging or AFO [49].

## Discussion

### Principal Findings

This review used a biomechanical framework to synthesize evidence on the interacting effects of footwear parameters and AFOs on gait outcomes in patients and healthy young adults. While it is evident that footwear adaptations can optimize gait biomechanics beyond what can be achieved by an AFO and standard shoe alone, large knowledge gaps remain, preventing empirical validation of many theoretical and biomechanical assumptions and adoption in clinical practice. The effects of plantar pressure [39,41] will be omitted from the discussion to focus on kinetics and kinematics.

Participants both with and without pathologies were included in this review, as healthy walkers can provide a valuable model for the biomechanical effects of AFO-FCs. Indeed, in the few studies that examined the same AFO type, footwear adaptation, and outcome measure, it was found that both patients after stroke and healthy walkers have increased knee flexion during the second rocker when the HSD is increased [38,43,45]*.* However, this heterogeneity in participants does require discretion when interpreting the results, as adaptations may not be transferable to all patient populations.

A wide range of AFO designs were included in this study; however, the majority (11/19) could be generalized as rigid AFOs which restrict both plantarflexion and dorsiflexion and can be assumed to have negligible differences in their mechanical properties when interpreting sagittal plane gait variables.

### Biomechanical Effects

#### Heel-Sole Differential

Adaptations increasing the HSD were comparatively well represented in this review, likely due to the SVA being considered a core component of the AFO tuning process [11,50]. Indeed, increasing the SVA, such as with a larger HSD, causes a cascade effect on lower limb kinematics and kinetics when the ankle joint is fixed. Notably, a larger HSD increased knee and hip flexion during the second rocker [45]. This increase in knee flexion was seen across all 3 papers that evaluated knee angle as a result of changing HSD in both healthy participants and patients after stroke [38,43,45]. Although a larger HSD was reported to increase ankle dorsiflexion despite the use of a rigid AFO, it should be noted that this increase may be due to marker placement on the shoe while using insoles to increase the HSD, causing an increase in SVA without a change in the participants’ ankle angle [45].

With a constrained ankle joint, changes in HSD affect both the knee and hip joints, indicating that shank kinematics are also influenced. This is supported by the study of Kerkum et al (2021) [7], which examined the dynamic SVA at midstance during walking with an AFO and found that, with a rigid AFO, the SVA increases as HSD increases [45]. Intuitively, the HSD can be used to influence lower limb joint kinematics in the first and second rockers; however, when the HSD is altered within the shoe, kinematics and kinetics at the ankle, knee, and hip joints are also influenced in the third rocker for rigid AFO users [47]. This is likely due to the fixation of the ankle joint, causing the shank to become more inclined as the foot-to-floor angle increases with a larger HSD, even as the heel leaves the ground in the third rocker. When the ankle joint is not constrained, it is able to adapt to changes in the HSD, leaving shank kinematics unaffected [21,51]. These findings suggest that patients who wear rigid AFOs and experience excessive knee extension may benefit from an increased HSD.

Ikeda et al (2018) [46] demonstrated that the ROS radius of curvature could be linearly increased with a constrained ankle and increasing HSD. Conversely, past research has shown that ROS are not affected by an increased HSD for unconstrained ankles [21]. As ROS radii between 20% and 30% leg length have been shown to optimize metabolic rate [15], the use of HSD to tune the ROS radius in rigid AFO users may be something to consider when optimizing energy consumption. Walking speed and cadence remained unaffected across different HSD settings, suggesting that while HSD can be used to manipulate joint kinetics and kinematics, it may have a limited impact on spatio-temporal parameters.

#### Heel Rocker

It was shown that a heel with a 20-degree bevel could reduce the knee extensor moment in the first rocker [28]. This follows biomechanical theory, which suggests that a heel rocker will reduce the moment arm of the GRF, thereby reducing the peak moments and angular velocities at the knee and ankle joints, facilitating smooth COM transfer. In other words, the use of a heel rocker could elicit a steeper ROS arc during first rocker, increasing the rate of forward progression of the COP. This suggests that such a heel rocker could be used to improve the walking efficiency of patients using rigid AFOs who struggle with step-to-step transitions or walk with excessive knee flexion at the start of the second rocker.

Changes in walking speed were not observed when a heel rocker was used with a rigid AFO [28]. As the other study that examined heel rockers did not look at temporal effects, no conclusion can be drawn based on the evidence presented in this review.

#### Heel Hardness

A more compliant heel was associated with reduced ankle dorsiflexor and knee extensor moments at initial contact, likely due to a shortened moment arm of the GRF [28]. Following biomechanical theory, it was seen that a more compliant heel had a similar effect to a rocker heel in terms of decreasing the knee extensor moment during the loading response [28,46]. The study by Wiest et al (1979) [28] demonstrated that a SACH could reduce the knee extensor moment during initial contact more than both a crepe heel and 20-degree beveled heel. As the SACH was constructed out of medium-density polyurethane, it may be more compressible and have greater force distribution properties than the crepe heel and incompressible heel, resulting in a smaller GRF moment arm at the knee and ankle joints at initial contact. The use of a compressible heel may therefore help reduce joint loading and smooth step-to-step transitions at initial contact for individuals with a heavy heel strike.

Increasing heel hardness was associated with increased peak dorsiflexor and knee extensor moments during the first rocker, as well as delayed peak moment timing [46]. These effects were most pronounced with wedges thicker than 1 cm, suggesting a threshold beyond which mechanical influence plays a greater role due to the increased vertical component of the GRF.

Ikeda et al [46] found that a more compliant heel placed the ROS center of curvature more posteriorly when compared to a stiffer heel. This is in line with the work of Hansen et al [52], which demonstrated that more compliant feet have a more curved ROS compared to less compressible prosthetic feet. This suggests that the use of a compressible heel wedge could be used to decrease the rate of COP forward progression during first rocker when using a rigid AFO, thereby increasing the steepness of the ROS arc. Using a more compliant heel may be a method of increasing the steepness of the ROS arc without introducing greater moments at the joint, such as with a harder heel. While harder heels were associated with greater gait speeds, they may also increase joint loading, which could be detrimental in populations with joint degeneration or pain [28]. When joint loading is not a concern, harder heels may be used to slightly increase gait speed due to the increased moments and angular momentum at the knee and ankle, leading to a faster COM transition between steps.

#### Forefoot Rocker

In patients after stroke, the use of a proximal forefoot rocker demonstrated differing effects depending on the type of AFO used. When paired with rigid AFOs, it increased knee flexion at toe-off as well as walking speed when compared to the same AFO with a standard shoe. This increase in walking speed may be due to improved rollover through the restoration of a more typical ROS radius caused by an increase in the COP velocity during third rocker. In line with this finding, it has been shown that a proximal forefoot rocker used without an AFO can facilitate improved weight transfer and a steeper ROS arc in healthy participants [25]. Increasing knee flexion at toe-off may also be beneficial in patients with limited foot clearance using rigid AFOs.

When combined with dorsiflexion-free AFOs, the peak ankle plantarflexor moment decreased when compared to the same AFO used with a standard shoe. This is likely due to the moment arm of the GRF being shortened by the proximal position of the rocker apex. These dorsiflexion-free AFOs were also shown to decrease stride length and walking speed when used with a proximal forefoot rocker instead of a standard shoe, possibly due to the reduced plantarflexor moment during third rocker.

#### Future Steps

A key finding of this review is the lack of research into varying rocker parameters such as both heel and forefoot apex positions and rocker radii when AFOs are used. The evidence from the limited studies included in this review suggests that rocker outsole parameters can have a significant impact on both healthy and pathological gait, indicating that further research into these areas could benefit AFO users. Of the 5 studies that used forefoot rockers, 4 studies used the same design [37,40,44,48], with limited reasoning behind their choice, and the fifth did not provide any descriptor of the rocker design [43]. It would be beneficial when using different AFOs with the same footwear adaptations to compare the same outcome measures, as this furthers the understanding of the complex interactions between AFOs and footwear combinations.

Future studies would benefit from studying the effects of differing forefoot rocker design choices, as these have been shown to have a significant impact on gait biomechanics [8,9,25]. In particular, understanding the biomechanical effects of differing apex positions and radii across a variety of AFO designs beyond rigid polypropylene AFOs would be beneficial. These parameters directly affect the GRF lever arms and shank kinematics, in addition to the timing of key gait events, and as such may have significant contributions to improving gait efficiency. A core understanding of the mechanisms driving biomechanical adaptations could lead to improvements in tuning AFOs to improve step-to-step transitions and minimize energy expenditure for patients with a range of pathologies.

An aim of this review was to consolidate evidence across diverse pathologies; however, the majority of included studies focused on patients after stroke and healthy participants. Broader representation would improve the generalizability of findings, as gait impairments in stroke differ from those in conditions such as cerebral palsy, multiple sclerosis, or peripheral neuropathy. Furthermore, focusing on a single pathology overlooks the considerable variability that exists within that group, potentially masking important individual differences. This narrow scope may hinder clinical decision-making, as practitioners often treat diverse patient populations and require evidence that supports personalized, condition-appropriate prescriptions. Future work should consider broadening the scope to include a wider range of pathologies in order to improve the clinical relevance and applicability of findings.

### Limitations

A very specific set of exclusion criteria was used for this review. While the number of included studies reflects similar reviews and the existing knowledge gap, a broader inclusion protocol could have been beneficial. For instance, the inclusion of papers that did not use an AFO for all conditions but had multiple footwear adaptations could still have provided insight into the effects different footwear adaptations may have on biomechanics. Furthermore, data were extracted by a single author, and while the included results were examined by the other 2 authors of this review, it is possible that some data were overlooked or inconsistently collected during the inclusion process, introducing a risk of subjective bias and potentially reducing the reproducibility of this review’s findings. As all studies included at least one significant finding, there may be a risk of publication bias. However, due to the design of the included studies, a formal assessment of publication bias was not performed.

While the papers included in this study were mostly of “good” quality, none could be considered “excellent.” This is largely due to no studies being blinded; however, due to the physical nature of the interventions, it would be difficult to truly blind participants and researchers. It is important to note that 4 of the 5 studies using forefoot rockers belong to the same institution. While each paper from that group reported different outcome measures, the same rocker parameters were used for all 4 papers.

A major limitation of this review is the lack of empirical descriptors given for interventions. Without empirical descriptors, it is difficult to draw scientifically sound conclusions from the presented outcomes. Many AFO details were inferred, or the details provided were too vague to derive conclusions from. For instance, 2 AFO designs were described as “more stiff” or “less stiff” than the other [41]. As AFO stiffness has a significant impact on knee and ankle kinematics, whether that difference in stiffness is 1 or 5 Nm/deg can have a substantial influence on gait outcomes [53]. Compounding this issue, very few studies gave sufficient detail about their adaptations, and there was often minimal description of the footwear being used. This lack of transparency makes it difficult to replicate findings or understand the specific mechanical contributions of the footwear-AFO combinations being tested.

### Conclusions

The results demonstrate that footwear adaptations can be used to optimize AFO effects for individual patients. However, while the biomechanical effects of increasing the HSD with a rigid AFO are well explored, there exists limited information regarding the effects of other outsole design parameters and compliant AFOs. Further understanding of the interactions between AFOs and rocker outsole parameters is required to maximize AFO effectiveness across different patient populations. Future research should investigate the effects of varying forefoot parameters, including rocker radius, apex position, and footplate stiffness, in combination with a wider range of AFO designs including energy-storing AFOs. Explicit and consistent reporting of AFO and footwear design properties is necessary to make meaningful conclusions on biomechanical outcomes.

## Appendix

Multimedia Appendix 1 Search strings.

Multimedia Appendix 2 Results per question for the Downs and Black Checklist and AFO reporting checklist. AFO: ankle-foot orthosis.

Multimedia Appendix 3 PRISMA checklist. Items 24b and 24c can be added during the revision process.

## Abbreviations

AFO: ankle-foot orthosis
AFO-FC: ankle-foot orthosis and footwear combination
COM: center of mass
COP: center of pressure
EMG: electromyography
GRF: ground reaction force
HSD: heel-sole differential
PROSPERO: International Prospective Register of Systematic Reviews
PRISMA: Preferred Reporting Items for Systematic Reviews and Meta-Analyses
ROS: roll-over shape
SACH: solid ankle cushion heel
SVA: shank-to-vertical angle

## References

[1] Perry J, Burnfield J (2024). Gait Analysis: Normal and Pathological Function.

[2] Demir YP, Suzuki T (2017). Neuromuscular diseases and rehabilitation. Neurological Physical Therapy.

[3] Ploeger HE, Bus SA, Brehm M, Nollet F (2014). Ankle-foot orthoses that restrict dorsiflexion improve walking in polio survivors with calf muscle weakness. Gait Posture.

[4] Malas BS (2011). What variables influence the ability of an AFO to improve function and when are they indicated?. Clin Orthop Relat Res.

[5] Daryabor A, Arazpour M, Aminian G (2018). Effect of different designs of ankle-foot orthoses on gait in patients with stroke: a systematic review. Gait Posture.

[6] Ploeger HE, Waterval NFJ, Nollet F, Bus SA, Brehm M (2019). Stiffness modification of two ankle-foot orthosis types to optimize gait in individuals with non-spastic calf muscle weakness - a proof-of-concept study. J Foot Ankle Res.

[7] Kerkum Y, Philippart W, Houdijk H (2021). The effects of footplate stiffness on push-off power when walking with posterior leaf spring ankle-foot orthoses. Clin Biomech (Bristol).

[8] van Kouwenhove L, Verkerke G, Postema K, Dekker R, Hijmans J (2021). Effect of different forefoot rocker radii on lower-limb joint biomechanics in healthy individuals. Gait Posture.

[9] Hutchins S, Bowker P, Geary N, Richards J (2009). The biomechanics and clinical efficacy of footwear adapted with rocker profiles—evidence in the literature. Foot (Edinb).

[10] Eddison N, Chockalingam N (2013). The effect of tuning ankle foot orthoses-footwear combination on the gait parameters of children with cerebral palsy. Prosthet Orthotics Int.

[11] Owen E (2010). The importance of being earnest about shank and thigh kinematics especially when using ankle-foot orthoses. Prosthet Orthotics Int.

[12] Sobhani S, Hijmans J, van den Heuvel E, Zwerver J, Dekker R, Postema K (2013). Biomechanics of slow running and walking with a rocker shoe. Gait Posture.

[13] Paton J, Hatton AL, Rome K, Kent B (2016). Effects of foot and ankle devices on balance, gait and falls in adults with sensory perception loss: a systematic review. JBI Database System Rev Implementations Rep.

[14] Wilkins RA, Chapman LS, Emmel JC, Flannery T, Chapman GJ, Walwyn REA, Redmond AC, Siddle HJ (2022). A systematic review and narrative synthesis of footwear and orthotic devices used in the management of ankle haemarthrosis and haemarthropathy in haemophilia. Haemophilia.

[15] Adamczyk PG, Collins SH, Kuo AD (2006). The advantages of a rolling foot in human walking. J Exp Biol.

[16] Esposito ER, Ruble MD, Ikeda AJ, Wilken JM (2021). The effect of custom carbon ankle-foot orthosis alignment on roll-over shape and center of pressure velocity. Prosthet Orthotics Int.

[17] Hansen AH, Childress DS, Knox EH (2004). Roll-over shapes of human locomotor systems: effects of walking speed. Clin Biomech (Bristol).

[18] Hansen AH, Childress DS (2005). Effects of adding weight to the torso on roll-over characteristics of walking. J Rehabil Res Dev.

[19] Kuo AD, Donelan JM, Ruina A (2005). Energetic consequences of walking like an inverted pendulum: step-to-step transitions. Exerc Sport Sci Rev.

[20] Fatone S, Hansen AH (2007). Effect of ankle-foot orthosis on roll-over shape in adults with hemiplegia. J Rehabil Res Dev.

[21] Hansen AH, Childress DS (2004). Effects of shoe heel height on biologic rollover characteristics during walking. J Rehabil Res Dev.

[22] Waterval NFJ, Brehm MA, Harlaar J, Nollet F (2020). Description of orthotic properties and effect evaluation of ankle-foot orthoses in non-spastic calf muscle weakness. J Rehabil Med.

[23] Fatone S, Sorci E, Hansen A (2009). Effects of clinically prescribed ankle foot orthoses on ankle-foot roll-over shapes: a case series. JPO J Prosthetics Orthotics.

[24] Shang J, Geng X, Wang C, Chen L, Zhang C, Huang J, Wang X, Yan A, Ma X (2020). Influences of high-heeled shoe parameters on gait cycle, center of pressure trajectory, and plantar pressure in young females during treadmill walking. J Orthop Surg (Hong Kong).

[25] Forghany S, Nester CJ, Richards B, Hatton AL, Liu A (2014). Rollover footwear affects lower limb biomechanics during walking. Gait Posture.

[26] Singer ML, Kobayashi T, Lincoln LS, Orendurff MS, Foreman KB (2014). The effect of ankle-foot orthosis plantarflexion stiffness on ankle and knee joint kinematics and kinetics during first and second rockers of gait in individuals with stroke. Clin Biomech (Bristol).

[27] Waterval NFJ, Nollet F, Harlaar J, Brehm M (2019). Modifying ankle foot orthosis stiffness in patients with calf muscle weakness: gait responses on group and individual level. J Neuroeng Rehabil.

[28] Wiest DR, Waters RL, Bontrager EL, Quigley MJ (1979). The influence of heel design on a rigid ankle-foot orthosis. Orthotics Prosthetics.

[29] Torricelli D, Gonzalez J, Weckx M, Jiménez-Fabián R, Vanderborght B, Sartori M, Dosen S, Farina D, Lefeber D, Pons JL (2016). Human-like compliant locomotion: state of the art of robotic implementations. Bioinspiration Biomimetics.

[30] Brown D, Wertsch JJ, Harris GF, Klein J, Janisse D (2004). Effect of rocker soles on plantar pressures. Arch Phys Med Rehabil.

[31] Greve C, Schuitema D, Otten B, van Kouwenhove L, Verhaar E, Postema K, Dekker R, Hijmans JM (2019). Biomechanical effects of rocker shoes on plantar aponeurosis strain in patients with plantar fasciitis and healthy controls. PLoS One.

[32] Page MJ, McKenzie JE, Bossuyt PM, Boutron I, Hoffmann TC, Mulrow CD, Shamseer L, Tetzlaff JM, Akl EA, Brennan SE, Chou R, Glanville J, Grimshaw JM, Hróbjartsson A, Lalu MM, Li T, Loder EW, Mayo-Wilson E, McDonald S, McGuinness LA, Stewart LA, Thomas J, Tricco AC, Welch VA, Whiting P, Moher D (2021). The PRISMA 2020 statement: an updated guideline for reporting systematic reviews. BMJ.

[33] Halman A, Oshlack A (2024). Catchii: empowering literature review screening in healthcare. Res Synth Methods.

[34] Downs SH, Black N (1998). The feasibility of creating a checklist for the assessment of the methodological quality both of randomised and non-randomised studies of health care interventions. J Epidemiol Community Health.

[35] Hooper P, Jutai JW, Strong G, Russell-Minda E (2008). Age-related macular degeneration and low-vision rehabilitation: a systematic review. Can J Ophthalmol.

[36] Ridgewell E, Dobson F, Bach T, Baker R (2010). A systematic review to determine best practice reporting guidelines for AFO interventions in studies involving children with cerebral palsy. Prosthet Orthotics Int.

[37] Farmani F, Mohseni-Bandpei M, Bahramizadeh M, Aminian G, Abdoli A, Sadeghi-Goghari M (2016). The influence of rocker bar ankle foot orthosis on gait in patients with chronic hemiplegia. J Stroke Cerebrovasc Dis.

[38] Sutdet P, Polhan N, Prakotmongkol V, Poomulna J, Trifani F, Iswana T, Nipuni L, De Silva M, Tharin S (2019). The effect of tibia inclination on gait pattern of stroke patients with genu recurvatum who wear an ankle foot orthosis: a pilot study. Technol Disabil.

[39] Dhalla R, Johnson JE, Engsberg J (2003). Can the use of a terminal device augment plantar pressure reduction with a total contact cast?. Foot Ankle Int.

[40] Farmani F, Bandpei MAM, Bahramizadeh M, Aminian G, Nikoo MR, Sadeghi-Goghari M (2016). The effect of different shoes on functional mobility and energy expenditure in post-stroke hemiplegic patients using ankle-foot orthosis. Prosthet Orthotics Int.

[41] Kearney RS, Lamb SE, Achten J, Parsons NR, Costa ML (2011). In-shoe plantar pressures within ankle-foot orthoses: implications for the management of achilles tendon ruptures. Am J Sports Med.

[42] Arvin M, Kamyab M, Moradi V, Hajiaghaei B, Maroufi N (2013). Influence of modified solid ankle-foot orthosis to be used with and without shoe on dynamic balance and gait characteristic in asymptomatic people. Prosthet Orthotics Int.

[43] Jagadamma KC, Owen E, Coutts FJ, Herman J, Yirrell J, Mercer TH, Van Der Linden ML (2010). The effects of tuning an ankle-foot orthosis footwear combination on kinematics and kinetics of the knee joint of an adult with hemiplegia. Prosthet Orthotics Int.

[44] Masoumi S, Aminian G, Daryabor A (2020). The effects on functional balance in hemiplegic stroke individuals wearing an ankle foot orthosis with rocker bottom shoes. J Rehabil Sci Res (JRSR).

[45] Kerkum YL, Houdijk H, Brehm M, Buizer AI, Kessels ML, Sterk A, van den Noort JC, Harlaar J (2015). The shank-to-vertical-angle as a parameter to evaluate tuning of ankle-foot orthoses. Gait Posture.

[46] Ikeda AJ, Fergason JR, Wilken JM (2018). Effects of altering heel wedge properties on gait with the intrepid dynamic exoskeletal orthosis. Prosthet Orthotics Int.

[47] Choi H, Bjornson K, Fatone S, Steele KM (2016). Using musculoskeletal modeling to evaluate the effect of ankle foot orthosis tuning on musculotendon dynamics: a case study. Disabil Rehabil Assist Technol.

[48] Daryabor A, Aminian G, Arazpour M, Baniasad M, Yamamoto S (2021). The effects of ankle-foot orthoses with plantar flexion stop and plantar flexion resistance using rocker-sole shoes on stroke gait: a randomized-controlled trial. Turk J Phys Med Rehabil.

[49] Schmalz T, Blumentritt S, Drewitz H, Freslier M (2006). The influence of sole wedges on frontal plane knee kinetics, in isolation and in combination with representative rigid and semi-rigid ankle-foot-orthoses. Clin Biomech (Bristol).

[50] Eddison N, Chockalingam N, Osborne S (2015). Ankle foot orthosis-footwear combination tuning: an investigation into common clinical practice in the United Kingdom. Prosthet Orthotics Int.

[51] Owen E, Fatone S, Hansen A (2018). Effect of walking in footwear with varying heel sole differentials on shank and foot segment kinematics. Prosthet Orthotics Int.

[52] Hansen AH, Childress DS, Knox EH (2000). Prosthetic foot roll-over shapes with implications for alignment of trans-tibial prostheses. Prosthet Orthotics Int.

[53] Totah D, Menon M, Jones-Hershinow C, Barton K, Gates DH (2019). The impact of ankle-foot orthosis stiffness on gait: a systematic literature review. Gait Posture.

