# Supplementary material for: Ankle-Foot Orthosis and Footwear Interactions in Adult Healthy and Patient Populations: Systematic Review of Biomechanical Outcomes
Source: JMIR Rehabil Assist Technol. 2026 Oct 13;13:e93423. doi: 10.2196/93423 (PMC13714521; doi:10.2196/93423)
Supplement: Supplementary file 2 [file rehab_v13i1e93423_app1.docx]

**Scopus**

TITLE-ABS-KEY((“ankle foot ortho*” OR "orthotic device" OR AFO OR “AFO-FC” OR (ankle AND (orthotic* OR orthosis OR orthoses OR splint))) AND

(biomech* OR gait OR “gait analys*” OR locomotion OR walk* OR kinemat* OR tuning OR manipulation) AND

(shoe* OR footwear OR rocker* OR “roll-over” OR insert* OR wedge* OR tuning))

**Embase**

('ankle foot orthosis'/exp OR 'orthosis'/exp OR (“ankle foot ortho*” OR "orthotic device" OR afo OR “afo fc” OR (ankle AND (orthotic* OR orthosis OR orthoses OR splint))):ab,ti,kw) AND

('biomechanics'/exp OR 'gait'/exp OR 'walking'/exp OR (biomech* OR gait OR “gait analys*” OR locomotion OR walk* OR kinemat* OR tuning OR manipulation):ab,ti,kw)

AND ('shoe'/exp OR (shoe* OR footwear OR rocker* OR “roll-over” OR insert* OR wedge* OR tuning):ab,ti,kw)

**PubMed**

("Orthotic Devices"[Mesh] OR “ankle foot ortho*”[tiab] OR "orthotic device"[tiab] OR AFO[tiab] OR AFO-FC[tiab] OR (ankle[tiab] AND (orthotic*[tiab] OR orthosis[tiab] OR orthoses[tiab] OR splint[tiab]))) AND

("Gait"[Mesh] OR "Walking"[Mesh] OR "Biomechanical Phenomena"[Mesh] OR biomech*[tiab] OR gait[tiab] OR “gait analys*”[tiab] OR locomotion[tiab] OR walk*[tiab] OR kinemat*[tiab] OR tuning[tiab] OR manipulation[tiab]) AND

("Shoes"[Mesh] OR shoe*[tiab] OR footwear[tiab] OR rocker*[tiab] OR “roll-over” [tiab] OR insert*[tiab] OR wedge*[tiab] OR tuning[tiab])

**Web of Science**

(TS=("ankle foot ortho*" OR "orthotic device" OR AFO OR AFO-FC OR (ankle AND (orthotic* OR orthosis OR orthoses OR splint))) OR KP=("ankle foot orthosis" OR AFO OR "orthotic device")) AND
(TS=(biomech* OR gait OR “gait analys*” OR locomotion OR walk* OR kinemat* OR tuning OR manipulation) OR KP=(Biomechanics OR walking OR locomotion OR gait OR “gait analysis”)) AND
(TS=(shoe* OR footwear OR rocker* OR “roll-over” OR insert* OR wedge* OR tuning) OR KP=(Shoe))
