# Supplementary material for: Ankle-Foot Orthosis and Footwear Interactions in Adult Healthy and Patient Populations: Systematic Review of Biomechanical Outcomes
Source: JMIR Rehabil Assist Technol. 2026 Oct 13;13:e93423. doi: 10.2196/93423 (PMC13714521; doi:10.2196/93423)
Supplement: Supplementary file 3 [file rehab_v13i1e93423_app2.docx]

**Table S1.** Downs and Black checklist scored per question.

| Reference | 27 | 36 | 37 | 38 | 39 | 40 | 41 | 42 | 43 | 44 | 45 | 46 | 47 | 48 |
| --- | --- | --- | --- | --- | --- | --- | --- | --- | --- | --- | --- | --- | --- | --- |
| TOTAL | **17** | **19** | **18** | **16** | **19** | **18** | **18** | **17** | **19** | **19** | **16** | **14** | **16** | **17** |
| Q. 1 | 1 | 1 | 1 | 1 | 1 | 1 | 1 | 1 | 1 | 1 | 1 | 1 | 0 | 1 |
| Q. 2 | 1 | 1 | 0 | 1 | 1 | 1 | 1 | 1 | 1 | 1 | 1 | 1 | 1 | 1 |
| Q. 3 | 1 | 1 | 1 | 0 | 1 | 1 | 1 | 1 | 1 | 1 | 1 | 1 | 1 | 1 |
| Q. 4 | 1 | 1 | 1 | 1 | 1 | 1 | 1 | 1 | 1 | 1 | 1 | 1 | 1 | 1 |
| Q. 5 | 2 | 2 | 2 | 2 | 0 | 2 | 2 | 2 | 2 | 2 | 2 | 1 | 0 | 2 |
| Q. 6 | 1 | 1 | 1 | 0 | 1 | 0 | 1 | 1 | 1 | 1 | 1 | 1 | 1 | 1 |
| Q. 7 | 1 | 1 | 1 | 0 | 1 | 0 | 1 | 1 | 1 | 1 | 1 | 0 | 1 | 1 |
| Q. 8 | 0 | 0 | 0 | 0 | 0 | 0 | 0 | 0 | 0 | 0 | 0 | 0 | 0 | 0 |
| Q. 10 | 0 | 1 | 1 | 1 | 1 | 1 | 1 | 0 | 1 | 1 | 1 | 0 | 1 | 0 |
| Q. 11 | 1 | 1 | 1 | 1 | 1 | 1 | 1 | 0 | 1 | 1 | 1 | 0 | 0 | 0 |
| Q. 12 | 1 | 1 | 1 | 1 | 1 | 1 | 1 | 1 | 1 | 1 | 0 | 1 | 1 | 1 |
| Q. 13 | 0 | 1 | 1 | 1 | 1 | 1 | 0 | 1 | 0 | 1 | 0 | 1 | 1 | 1 |
| Q. 14 | 0 | 0 | 0 | 0 | 0 | 0 | 0 | 0 | 0 | 0 | 0 | 0 | 0 | 0 |
| Q. 15 | 0 | 0 | 0 | 0 | 0 | 0 | 0 | 0 | 0 | 0 | 0 | 0 | 0 | 0 |
| Q. 16 | 1 | 1 | 1 | 1 | 1 | 1 | 1 | 1 | 1 | 1 | 1 | 1 | 1 | 1 |
| Q. 18 | 1 | 1 | 1 | 1 | 1 | 1 | 1 | 1 | 1 | 1 | 1 | 1 | 1 | 1 |
| Q. 19 | 1 | 1 | 1 | 1 | 1 | 1 | 1 | 1 | 1 | 1 | 1 | 1 | 1 | 1 |
| Q. 20 | 1 | 1 | 1 | 1 | 1 | 1 | 1 | 1 | 1 | 1 | 1 | 1 | 1 | 1 |
| Q. 21 | 1 | 1 | 1 | 1 | 1 | 1 | 1 | 1 | 1 | 1 | 0 | 1 | 1 | 1 |
| Q. 22 | 1 | 1 | 1 | 1 | 1 | 1 | 1 | 1 | 1 | 1 | 0 | 1 | 1 | 1 |
| Q. 23 | 0 | 0 | 0 | 0 | 1 | 0 | 0 | 0 | 0 | 0 | 0 | 0 | 1 | 0 |
| Q. 24 | 0 | 0 | 0 | 0 | 0 | 0 | 0 | 0 | 0 | 0 | 0 | 0 | 0 | 0 |
| Q. 25 | 1 | 1 | 1 | 1 | 1 | 1 | 1 | 1 | 1 | 1 | 1 | 0 | 1 | 1 |
| Q. 27 | 0 | 0 | 0 | 0 | 1 | 1 | 0 | 0 | 1 | 0 | 1 | 0 | 0 | 0 |

Each question is scored binomially except for Q5 which is scored out of 2.

**Table S2.** AFO reporting checklist results.

| Authors | AFO details | | | | | | | | | Testing protocol | | |
| --- | --- | --- | --- | --- | --- | --- | --- | --- | --- | --- | --- | --- |
|  | **Suitability** | **Movements** | **AFO ankle angle** | **Materials** | **Trimlines** | **Tuning** | **Shank-to-vertical angle** | **Mechanical properties** | **Manufacture** | **Control** | **Order of testing** | **Acclimatization** |
| Farmani et al. 2016 [36] | Clear | Complete | Specified for group | Complete | Some clear | Not stated | Not stated | Partial | Custom | Barefoot | Randomized | <1 day |
| Sutdet et al. 2019 [37] | Clear | Complete | Specified for group | Complete | Not stated | Complete | Complete | Partial | Custom | Shoes | Not stated | NA |
| Dhalla et al. 2003 [38] | Clear | Complete | Specified for group | Complete | Some clear | Partial | Not stated | Not stated | Custom | Shoes | Not stated | Not stated |
| Farmani et al. 2016 [39] | Clear | Incomplete | Specified for group | Partial | Not stated | Partial | Not stated | Partial | Prefabricated | Shoes | Randomized | Not stated |
| Kearney et al. 2011 [40] | Clear | Incomplete | Not stated | Complete | All clear | Not stated | Not stated | Not stated | Prefabricated | Shoes | Randomized | Not stated |
| Kearney et al. 2011 [40] | Clear | Incomplete | Not stated | Complete | All clear | Not stated | Not stated | Not stated | Prefabricated | Shoes | Randomized | Not stated |
| Arvin et al. 2013 [41] | Clear | Complete | Specified for group | Complete | All clear | Not stated | Not stated | Not stated | Custom | Not stated | Randomized | Not stated |
| Jagadamma et al. 2010 [42] | Clear | Incomplete | Specified for individual | Complete | Some clear | Complete | Complete | Not stated | Custom | Not stated | Non-randomized | NA |
| Masoumi et al. 2020 [43] | Clear | Incomplete | Ambiguous | Complete | Some clear | Partial | Not stated | Not stated | Custom | Shoes | Randomized | 1-4 weeks |
| Kerkum et al. 2015 [44] | Clear | Complete | Specified for group | Partial | Some clear | Complete | Complete | Complete | Prefabricated | Shoes | Randomized | <1 day |
| Wiest et al. 1979 [27] | Clear | Complete | Specified for group | Partial | Some clear | Not stated | Not stated | Partial | Custom | Not stated | Randomized | <1 day |
| Ikeda et al. 2018 [45] | Clear | Complete | Ambiguous | Complete | All clear | Complete | Not stated | Complete | Prefabricated | Not stated | Randomized | 1-4 weeks |
| Choi et al. 2016 [46] | Clear | Incomplete | Ambiguous | Not stated | Not stated | NA | Not stated | Not stated | Prefabricated | Shoes | Non-randomized | Not stated |
| Choi et al. 2016 [46] | Clear | Complete | Specified for individual | Partial | All clear | Complete | Complete | Partial | Custom | Shoes | Non-randomized | > 4 weeks |
| Daryabor et al. 2021 [47] | Clear | Complete | Specified for group | Partial | Some clear | Complete | Not stated | Partial | Custom | Shoes | Randomized | 1-4 weeks |
| Daryabor et al. 2021 [47] | Clear | Complete | Specified for group | Partial | Some clear | Complete | Not stated | Partial | Custom | Shoes | Randomized | 1-4 weeks |
| Schmalz et al. 2006 [48] | Clear | Complete | Not stated | Not stated | All clear | Complete | Not stated | Not stated | Prefabricated | Shoes | Randomized | Not stated |
| Schmalz et al. 2006 [48] | Clear | Complete | Not stated | Not stated | Some clear | Complete | Not stated | Not stated | Not stated | Shoes | Randomized | Not stated |

**References:**

27. Waterval NFJ, Nollet F, Harlaar J, Brehm M. Modifying ankle foot orthosis stiffness in patients with calf muscle weakness: gait responses on group and individual level. J Neuroeng Rehabil. 2019;16(1):120. [FREE Full text] [doi: 10.1186/s12984-019-0600-2] [Medline: 31623670]

36. Ridgewell E, Dobson F, Bach T, Baker R. A systematic review to determine best practice reporting guidelines for AFO interventions in studies involving children with cerebral palsy. Prosthet Orthotics Int. 2010;34(2):129-145. [doi: 10.3109/03093641003674288] [Medline: 20384548]

37. Farmani F, Mohseni-Bandpei M, Bahramizadeh M, Aminian G, Abdoli A, Sadeghi-Goghari M. The influence of rocker bar ankle foot orthosis on gait in patients with chronic hemiplegia. J Stroke Cerebrovasc Dis. 2016;25(8):2078-2082. [doi: 10.1016/j.jstrokecerebrovasdis.2016.02.021] [Medline: 27265095]

38. Sutdet P, Polhan N, Prakotmongkol V, Poomulna J, Trifani F, Iswana T, et al. The effect of tibia inclination on gait pattern of stroke patients with genu recurvatum who wear an ankle foot orthosis: a pilot study. Technol Disability. 2019;31(3):77-82. [doi: 10.3233/tad-180211]

39. Dhalla R, Johnson JE, Engsberg J. Can the use of a terminal device augment plantar pressure reduction with a total contact cast? Foot Ankle Int. 2003;24(6):500-505. [doi: 10.1177/107110070302400609] [Medline: 12854672]

40. Farmani F, Bandpei MAM, Bahramizadeh M, Aminian G, Nikoo MR, Sadeghi-Goghari M. The effect of different shoes on functional mobility and energy expenditure in post-stroke hemiplegic patients using ankle-foot orthosis. Prosthet Orthotics Int. 2016;40(5):591-597. [doi: 10.1177/0309364615592704] [Medline: 26184035]

41. Kearney RS, Lamb SE, Achten J, Parsons NR, Costa ML. In-shoe plantar pressures within ankle-foot orthoses: implications for the management of achilles tendon ruptures. Am J Sports Med. 2011;39(12):2679-2685. [doi: 10.1177/0363546511420809] [Medline: 21908718]

42. Arvin M, Kamyab M, Moradi V, Hajiaghaei B, Maroufi N. Influence of modified solid ankle-foot orthosis to be used with and without shoe on dynamic balance and gait characteristic in asymptomatic people. Prosthet Orthotics Int. 2013;37(2):145-151. [doi: 10.1177/0309364612454159] [Medline: 22907949]

43. Jagadamma KC, Owen E, Coutts FJ, Herman J, Yirrell J, Mercer TH, et al. The effects of tuning an ankle-foot orthosis footwear combination on kinematics and kinetics of the knee joint of an adult with hemiplegia. Prosthet Orthotics Int. 2010;34(3):270-276. [doi: 10.3109/03093646.2010.503225] [Medline: 20738231]

44. Masoumi S, Aminian G, Daryabor A. The effects on functional balance in hemiplegic stroke individuals wearing an ankle foot orthosis with rocker bottom shoes. J Rehabil Sci Res (JRSR). 2020;7(3):141-145. [doi: 10.30476/jrsr.2020.86778.1094]

45. Kerkum YL, Houdijk H, Brehm M, Buizer AI, Kessels ML, Sterk A, et al. The shank-to-vertical-angle as a parameter to evaluate tuning of ankle-foot orthoses. Gait Posture. 2015;42(3):269-274. [doi: 10.1016/j.gaitpost.2015.05.016] [Medline: 26050873]

46. Ikeda AJ, Fergason JR, Wilken JM. Effects of altering heel wedge properties on gait with the intrepid dynamic exoskeletal orthosis. Prosthet Orthotics Int. 2018;42(3):265-274. [doi: 10.1177/0309364617728116] [Medline: 28870146]

47. Choi H, Bjornson K, Fatone S, Steele KM. Using musculoskeletal modeling to evaluate the effect of ankle foot orthosis tuning on musculotendon dynamics: a case study. Disabil Rehabil Assist Technol. 2016;11(7):613-618. [FREE Full text] [doi: 10.3109/17483107.2015.1005030] [Medline: 25640240]

48. Daryabor A, Aminian G, Arazpour M, Baniasad M, Yamamoto S. The effects of ankle-foot orthoses with plantar flexion stop and plantar flexion resistance using rocker-sole shoes on stroke gait: a randomized-controlled trial. Turk J Phys Med Rehabil. 2021;67(4):449-461. [FREE Full text] [doi: 10.5606/tftrd.2021.6448] [Medline: 35141485]
